# Detection of *Bartonella vinsonii*, *Anaplasma platys* and *Bartonella* sp. in *didelphis marsupialis*, *Pecari tajacu* and *Chelonoidis denticulate*: Peru

**DOI:** 10.1186/s13104-023-06412-0

**Published:** 2023-07-20

**Authors:** Jesús Rojas-Jaimes, Juana del Valle-Mendoza

**Affiliations:** 1 Facultad de Ciencias de la Salud, Universidad Privada del Norte, Av. El Sol 461, San Juan de Lurigancho 15434, Lima, Peru; 2 Escuela de Medicina Humana, Universidad Peruana de Ciencias Aplicadas, Lima, Peru

**Keywords:** *Bartonella vinsonii*, *Anaplasma platys*, *Didelphis marsupialis*, *Pecari tajacu*, *Chelonoides denticulata*

## Abstract

**Introduction:**

Evidence suggest that wildlife Infectious diseases related to wildlife are of most importance because of the agents’ capacity to spill over into humans from the wild reservoir. Among them, the bacteria *Bartonella* spp. and *Anaplasma* spp. are related to this zoonotic dynamic.

**Objective:**

The primary goal of the present study was to determine the presence of pathogenic bacteria in kidney and liver tissues of *Didelphis marsupialis*; spleen, liver, and skin of *Pecari tajacu*; spleen, liver, and skin of *Chelonoidis denticulata*.

**Methodology:**

A PCR using universal and specific primers for 16 S rRNA, of *Bartonella spp*. with subsequent genetic sequencing were used.

**Results:**

The results in this study indicate that *Bartonella vinsonni* was detected in the liver tissue of *Didelphis marsupialis* using both universal primers and those specific for *Bartonella* sp. *Anaplasma platys* was detected at the liver and spleen level using universal primers. Additionally, *Bartonella spp.* was found at the liver, spleen, and skin level in *Pecari tajacu* using the specific primers. Finally, using the universal and specific primers at the skin level, *Bartonella* spp. was evident in *Chelonoidis denticulata.*

**Conclusions:**

The presence of the DNA of the *Bartonella vinsonii* was detected at the liver tissue in *Didelphis marsupialis*. DNA of the *Anaplasma platys* and *Bartonella spp.* were identified at the spleen and liver level. This study also identified that DNA *Bartonella spp.* was detected in *Pecari tajacu* skin. Finally DNA of *Bartonella* spp. was evident in *Chelonoidis denticulate* skin. The findings of this study suggest that these bacteria are present in these animals and may be responsible for outbreaks.

## Introduction

Human activities play critical role in the infectious diseases originating in wildlife. Previous studies have found that human and domestic animal intrusion impact in wildlife ecology [1, 2]. The same study found that an average of 60% of emerging infectious diseases were zoonotic, including bacteria such as *Bartonella sp.* and *Anaplasma* sp. Moreover, there is evidence that these bacteria can be transmitted from different animals to humans [3].

*Bartonella* is a bacterial genus that involves some species that are pathogenic for humans, such as *Bartonella bacilliformis, Bartonella henselae, and Bartonella quintana*, as well as others that are potentially pathogenic, including *Bartonella elizabethae, Bartonella tamiae*, and *Bartonella vinsonii*, subspecies *aurapensis* [4, 5]. It has previously been detected that these are causative agents of clinical manifestations in humans (4–6). In 1999 *B. vinsonii* subsp. *arupensis* was first isolated in a case of bacteremia in a rancher, in Wyoming-USA [6], and has been described in rodents such as *Peromyscus maniculatus.* This bacterium is an important component for public health because it carries hantavirus which can be transmitted to humans [7]. Additionally, evidence suggests that *B. vinsonii* subsp. *Arupensis* is a causative agent of endocarditis and febrile processes in humans and dogs [8–13].

*Anaplasma* is a proteobacterium that multiplies at the intracellular level is widely distributed in tropical, subtropical and temperate regions. A number of studies have postulated that *Anaplasma* can cause anaplasmosis in variety of animals including ruminants, rodents, birds, and humans [14–16]. Detailed examination of this proteobacterium showed it can invade blood cells of mammals such as erythrocytes, leukocytes, and thrombocytes [17]. Four species of *Anaplasma* sp (*Anaplasma phagocytophilum, Anaplasma capra, Anaplasma platys* and *Anaplasma ovis*). are zoonotic pathogens and are transmitted by ticks generating a problem for both Veterinary and Public Health [17].

## Main text

### Methods

#### Sampling

**Fig. 1:**
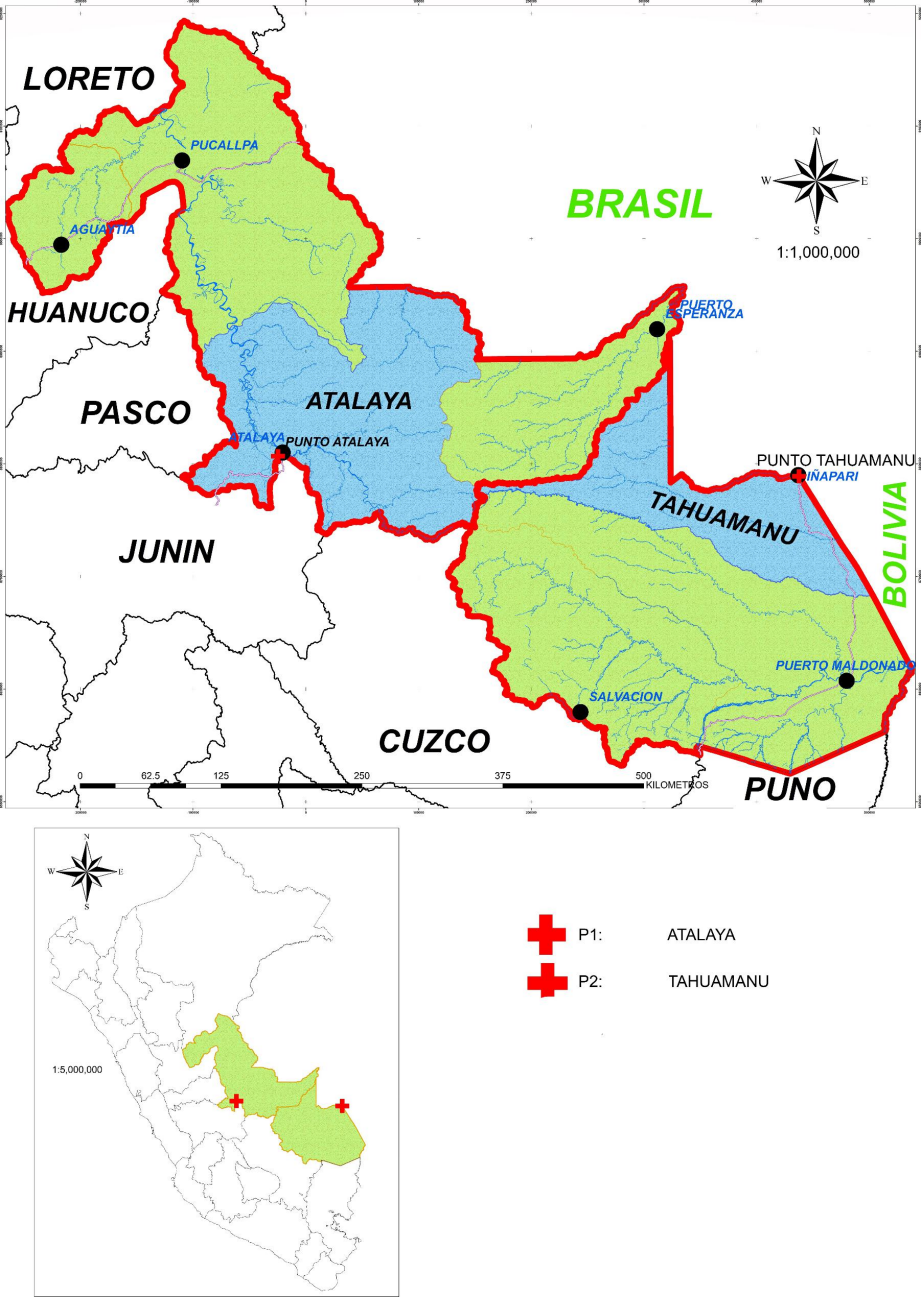
Location of the sites where tissue samples were collected in the provinces of Tahuamanu in Madre de Dios (*P. tajacu* and *C. denticulata*) and Atalaya in Ucayali, in Peru, (*D. marsupialis*). This map was created with the Geoserver https://geoservidor.minam.gob.pe/ edited with ArcGis 10.3.1 version 2015

A total sample of six were collected in Tahuamanu (10°57′16″S 69°34′37″W, Madre de Dios, Peru). The sample were taken from the liver, spleen, and skin of *Pecari tajacu* and *Chelonoidis denticulata*. Three samples of *Didelphis marsupialis* were collected from Atalaya (10°44′00″S 73°45′00″W, Ucayali, Peru) (Fig. 1). The samples were from the liver, spleen and kidney. Once the samples were extracted, they were shipped back to the molecular laboratory at Universidad Peruana de Ciencias Aplicadas for processing.

#### DNA extraction

Previously, 50 ug of tissue was digested using 200 µl of lysis buffer (proteinase K 20 mg/ml, 50 µL, 1 M Tris-HCl solution 10 µL, 0.5 M EDTA 2 µL, 10% SDS 100 µL, and distilled water 838 ml), and was incubated at 52 °C overnight until all tissue fragments were completely dissolved. Further extraction and purification procedures were performed using a commercial extraction kit (High Pure Template Preparation Kit, Roche Applied Science®, Mannheim, Germany). The bacterial DNA obtained after extraction was diluted in 100 µL of nuclease-free water and, then, processed or stored at -20 °C until use.

#### PCR procedures

Two different PCR approaches for 16 S rRNA were used: one with specific primers for *Bartonella* genus and the other with universal primers. All samples were used with specific primers for Bartonella as universal.

#### Amplification of fragment from the 16 S rRNA gene specific to the Bartonella spp

A 438-bp fragment of the 16 S rRNA gene specific to the *Bartonella* genus was amplified in blood samples (P24Emod CCTTCAGTTMGGCTGGATC- 16 S-R GCCYCCTTGCGGTTAGCACA) [18, 19]. The amplified products were in-gel recovered, purified using the SpinPrep™ Gel DNA Kit® (EMD Biosciences, Madison, WI, USA), and submitted for sequencing (Macrogen, Seoul, Korea).

#### Amplification of 16 S rRNA gene fragments using universal primers

In the case of amplifying any bacteria, the molecular diagnosis was confirmed by amplification and sequencing of a 1503-bp region of universal 16 S rRNA gene fragments using universal primers (8 F AGAGTTTGATCCTGGCTCAG- 1510R GGTTACCTTTGTTACGACTT) [19, 20]. All products obtained were recovered and sequenced (Macrogen, Seoul, Korea).

#### Data analysis

DNA sequences were analyzed using the BLAST analysis tool and compared with the GenBank database.

## Results

All samples were analyzed by molecular techniques. Table 1 shows the correct amplification of 1,503 bp using universal 16 S rRNA primers in some tissues. This table also showed the presence of *B. vinsonni* in the spleen of *D. marsupialis* “Opossum” (1 case), *Bartonella* spp in the skin of *C. denticulata* “Yellow-footed tortoise” (1 case), and *Anaplasma* spp in the spleen and liver of *P. tajacu* (1 case).

To determine the presence of *Bartonella* spp., PCR approaches of the 16 S rRNA gene specific to the *Bartonella* were performed on all samples. Sequence analysis showed the presence *Bartonella vinsonni*-positive sample in the spleen of *D. marsupialis*. Further analysis showed that *Bartonella* spp. was identified in the liver, spleen and skin in *P. tajacu*. However, *Bartonella* spp. was only observed in the skin of *C. denticulata* (Table 1).

**Table 1:** Pathogens identified by PCR followed by automated sequencing in tissue samples

Specimen	Organ	Universal 16 S rRNA	*Bartonella* spp. *16 S rRNA* gene
*Didelphis marsupialis* (Opossum)	Spleen	*Bartonella vinsonni*	*Bartonella vinsonni*
	Liver	Negative	Negative
	Kidney	Negative	Negative
*Pecari tajacu* (*Sajino*)	Spleen	*Anaplasma platys*	*Bartonella* spp.
	Liver	*Anaplasma platys*	*Bartonella* spp.
	Skin	Negative	*Bartonella* spp.
*Chelonoidis denticulata* (Yellow-footed tortoise)	Spleen	*Negative*	*Negative*
	Liver	Negative	Negative
	Skin	*Bartonella* spp	*Bartonella* spp.

## Discussion

In our study, *A. platys* was identified at the spleen and liver of *P. tajacu*. According to a previous study, *P. tajacu* was infested by ticks such as *Rhipicephalus* (*Boophilus*) *microplus* [21]. An implication of this finding is for veterinarians and public health. For instance a study in Venezuela found that *A. platys* was identified in *R. sanguineus* demonstrating the critical role of ticks in transmitting the bacterium *Anaplasma* sp. to the animals [22, 23]. These results corroborate the findings of previous studies where *A. platys* and *A. capra* were observed in *R. microplus* collected from goats, cattle, and sheep [17, 24]; These results are consistent with earlier studies where *A. platys* were found in dogs and *R. sanguineus* [17, 24]. Similarly, a recent report in Tunisia identified *A. phagocytophilum* in dogs, horses and ticks such as *Ixodes ricinus, Hyalomma scupense*, and *H. marginatum*. The same report found *Anaplasma* spp. and *A. ovis* in goats and sheep [25–28]. While in Palestine, *A. platys* was identified in dogs and *Anaplasma* sp. in sheep [29].

In South America, a study in Brazil identified *Anaplasma* spp. in animals of the *Xenarthra* superorder, specifically in *Bradypus tridactylus*, *B. variegatus*, *Choloepus didactylus, Tamandua tetradactyla, Myrmecophaga tridactyla, Cabassous unicinctus, Dasypus novemcinctus*, and *Euphractus sexcinctus*. Additionally another study showed the prevalence of *Anaplasma spp.* in several species of wild carnivorous animals, indicating the potential risk of the bacterium into humans whenever they encounter wild animals for tourism, consumption, tourism or other extractive activities [14, 30, 31]. In this study *Anaplasma* spp. was identified in the spleen and liver of *P. tajacu*, therefore this could be considered as reservoir. Further studies are necessary for detecting the bacteria in culture and elucidating the role of ectoparasites such as *R. microplus* ticks that were detected infesting *P. tajacu* [22].

In our study, *Bartonella sp.* was identified at the liver, spleen, and skin level in *P. tajacu*. The identification of *Bartonella* related to this animal is reinforced by the previous study in which *B. bacilliformis* was detected in the ectoparasites of *P. tajacu* such as *R. microplus* [32]. It can therefore be assumed that this animal is a potential reservoir, and it highlights the potential risk of infection by this bacterium through the bite of ticks on people in Tahuamanu and other rainforest, areas in Peru, where they hunt *P. tajacu* for consumption.

In the current study, *B. vinsonii* was found at the spleen level in *Didelphis marsupialis*. This animal could be involved in the zoonotic chain of this bacterium. For example *B. vinsonii* has been reported in animals such as *Canis latrans* “coyote” as a reservoir where this bacterium could generate endocarditis in humans [12, 13]. Another study also found *B. vinsonii* in carnivores such as *Canis latrans* “coyote”, *Vulpes vulpes* “Red Fox”, and *Procyon lotor* “Raccoon” [33]. In Peru, *B. v. berkhoffii* and *B. rochalimae* were found in asymptomatic domestic dogs, so they could be reservoirs of risk for human infection [34].

*Bartonella sp.* was also identified in the skin of *Chelonoidis denticulata*, which presumes that the bacterium can be transmitted to other animals through vectors such as ticks although the species was not determined to identify any potential risk of causing any disease in humans. However, it is known that the *Bartonella* genus can spill over into different animals, especially from rodents to humans and from rodents to domestic animals [35]. An implication of this result is the possibility of, prevention fields.

In conclusion, for the first time, we have been able to detect *B. vinsonni* in spleen from *D. marsupialis* and *A. platys* in liver and kidney from *P. tajacu*. These results are significant due to the risk of infection by these bacteria to people who hunt these wild animals for their meat. As well as the transmission of the infection to domestic animals in areas where the presence of wild animals studied generated bacterial zoonoses.

### Limitations

A limitation of this study is that, we can not assure the viavility of these bacteria. A further study should contemplate cultivating these bacteria of tissues such as skin to determine the role of these animals as reservoirs. Also the animals and samples in the present study were a reduced number.

## Data Availability

All data and materials used in the experiment are included in the article. Abstraction format used in the study and dataset are available and accessible from the below link. The link: https://figshare.com/articles/dataset/Wild/21383877?file=37952283.
